# The Midwives Bill

**Published:** 1902-03-22

**Authors:** 


### The Hospital

A Journal of

The Medical Sciences and Hospital Administration.

Vol. XXXI.—No. 808.] Edited by Sir Henry Burdett, K.C.B., and Solomon C. Smith, M.D., M.R.C.P. [March 22, 1902.

### THE MIDWIVES BILL.

Just as no man can be declared happy until his death, so no Bill before Parliament can be regarded as secure until it has actually received the Royal assent. There are for every Bill, nevertheless, certain stages of a critical character; and, when these have once been safely traversed, the probabilities of a successful issue become greatly enhanced. The passage of the Midwives Bill, with only inconsiderable amendments, through the Standing Committee on Law of the House of Commons, renders it extremely likely that its progress towards the statute book will not be subjected to any serious interruption, and that the year 1903 will find the women of this country in a position to distinguish, if they wish to do so, between midwives who have received instruction and those who have not. It is satisfactory to find that such thoroughly representative members of the medical profession as Sir Walter and Sir Michael Foster, and Sir John Batty Tuke, took active parts in the discussions in the Committee; and also that, by the admission of Mr. Heywood Johnstone, who had charge of the Bill, it is to be regarded as somewhat tentative in its character, and as such to be open to amendment whenever sufficient experience of its working has been obtained.

The chief amendments accepted by the Committee were the insertion of a prohibition of any description of a woman as a person "specially qualified to practise midwifery," by calling herself not midwife, but "accoucheuse, *sage-femme*, obstetric nurse, or other title, with the object of deceiving the public;" a provision that after the passing of the Act no woman should be able to recover a fee for services in the lying-in room unless she were duly certified; and an increase of the limit of the examination fee for a certificate from ten shillings to a guinea. The amendments rejected were endeavours to penalise the practice of uncertificated persons; to strike out the Royal College of Physicians of Ireland and the Obstetrical Society of London from the list of authorities qualified to certify to the Central Midwives' Board, or alternatively to include in that list a large number of maternity hospitals in London, Manchester, Birmingham, Liverpool, Edinburgh, Glasgow, and Dublin; to substitute the General Medical Council for the Lord President of the Privy Council as the authority for appointing two members of the Central Board, and for the medical corporations named for the purpose of appointing four such members; to provide that all seven of the members of the Board, instead of four only, should of necessity be medical practitioners, or (on the motion of Mr. T. P. O'Connor), that each of the four should be a man who holds, or has held, the appointment of teacher of midwifery at a recognised medical school; specially to exclude the Incorporated Midwives' Institute from the appointing authorities; to include the Royal British Nurses' Association; to include the Victoria University; and, finally, to impose a penalty upon a certified midwife for neglect to call in a medical man in any case of abnormality.

Alike in the acceptance and in the rejection of these several proposals, the Grand Committee appears to us to have exhibited an amount of common sense which is of good omen for the practical working of the measure. Perhaps the first place among the amendments in point of absurdity must be given to that proposed by Mr. T. P. O'Connor; because the best possible qualification for a seat on the Central Board will be an intimate knowledge of the practical requirements of poor urban or rural districts, such as present or past teachers of midwifery at recognised schools could scarcely by any possibility be expected to possess; while the natural tendency of these gentlemen would almost certainly be towards an undue and unnecessary increase of the requirements of the examination, which need not, in truth, be of a very elaborate character. Little more is required from such a midwife as the Bill contemplates than a knowledge of the supreme importance of cleanliness, of the risks of septic infection and of the way to obviate them, of the early signs of an abnormal presentation, or of extreme rigidity of the os uteri, of the utility of the unbroken membranes, and of the time when this utility ceases, and of the means of endeavouring to arrest an unexpected gush of hæmorrhage. In all probability the possession even of this amount of knowledge would render her cautious rather than rash. There was once a native of India, the descendant through several generations of a family that had "cut for the stone," whose brilliant skill and almost unbroken success as a lithotomist induced an English surgeon to give him instruction in the anatomy of the parts concerned in his operations, with the result that the poor man never ventured to take up his knife again. We fear that the teachers of midwifery might exert a not dissimilar influence upon the midwives. In the meanwhile, it seems manifest that the chief requisite for obtaining the best possible results from the Bill, if it should become law, will be the adoption of a proper organisation.

